# Statistical Study of Nonthermal Plasma-Assisted ZnO Coating of Cotton Fabric through Ultrasonic-Assisted Green Synthesis for Improved Self-Cleaning and Antimicrobial Properties

**DOI:** 10.3390/ma14226998

**Published:** 2021-11-18

**Authors:** Muhammad Irfan, Muhammad Y. Naz, Muhammad Saleem, Malik Tanawush, Adam Głowacz, Witold Glowacz, Saifur Rahman, Mater H. Mahnashi, Yahya S. Alqahtani, Bandar A. Alyami, Ali O. Alqarni, Mabkhoot A. Alsaiari

**Affiliations:** 1 Electrical Engineering Department, College of Engineering, Najran University Saudi Arabia, Najran 11001, Saudi Arabia; miditta@nu.edu.sa (M.I.); srrahman@nu.edu.sa (S.R.); 2 Department of Physics, University of Agriculture, Faisalabad 38040, Pakistan; saleem_192@yahoo.com (M.S.); imtanawush@gmail.com (M.T.); 3 Department of Automatic Control and Robotics, Faculty of Electrical Engineering, Automatics, Computer Science and Biomedical Engineering, AGH University of Science and Technology, al. A. Mickiewicza 30, 30-059 Kraków, Poland; adglow@agh.edu.pl (A.G.); wglowacz@agh.edu.pl (W.G.); 4 Department of Pharmaceutical Chemistry, College of Pharmacy, Najran University, Najran 11001, Saudi Arabia; matermaha@gmail.com (M.H.M.); yahyasalqahtani0@gmail.com (Y.S.A.); alyamibandar1@gmail.com (B.A.A.); aoqarni@gmail.com (A.O.A.); 5 Empty Qaurter Research Unit, Chemistry Department, College of Science and Art at Sharurah, Najran University Saudi Arabia, Najran 11001, Saudi Arabia; mabkhoot.alsaiari@gmail.com

**Keywords:** nonthermal plasma, *Psidium guajava*, zinc oxide, self-cleaning, antimicrobial activity, UV protection factor

## Abstract

Nonthermal plasma processing is a dry, environment-friendly and chemical-free method of improving the wettability, adhesion, self-cleaning and dying quality of fabrics without affecting their bulk properties. This study presents a green synthesis and coating method for the immobilization of nanoparticles of ZnO on the nonthermal plasma functionalized cotton fabric. The self-cleaning activity of ZnO-coated cotton was then optimized statistically. The ultraviolet protection and antimicrobial activity of the optimized and a control sample were also elaborated in this study. *Psidium guajava Linn* (guava) plant extract and zinc chloride were used in the ultrasonic biosynthesis of ZnO nanoparticles and concurrent immobilization over plasma functionalized cotton. Sodium hydroxide was used as a reaction accelerator. Statistical complete composite design (CCD) based on the amount of ZnCl_2_, NaOH and plasma exposure time was used to optimize the role of input parameters on the self-cleaning ability of the coated cotton. Methylene blue in water was used as a sample pollutant in the self-cleaning study. The ZnO-coated cotton showed notably high self-cleaning activity of 94% and a UV protection factor of 69.87. The antimicrobial activity against E. Coli and S. Aureus bacteria was also appreciably high compared to the control.

## 1. Introduction

Nanomaterials are known for large surface area to volume ratios. The applications of nanomaterials, especially in environmental settings, depend on the type and physicochemical properties of the material. Nanomaterials find their applications in the chemical industry, aerospace industry, optics, hydrogen fuel cell, sensors, batteries, power production devices, electronics, construction industry, thermoelectric devices, automotive engineering, textile industry, cosmetic industry, pharmaceutics, etc. [1]. For each application, the morphological parameters of the nanomaterials can be tuned by altering the chemical concentration, reaction conditions and method of synthesis. However, nanomaterials still lack a fundamental synthesis mechanism, stability in hostile environments, modeling factors, biocompatibility and recyclability [2].

Green synthesis of nanomaterials is known for its low cost, high reliability, sustainable procedure and eco-friendly nature. This method did not produce any harmful byproducts, but the use of natural resources and ideal solvent systems is essential to meet this objective. The natural biological systems for the green synthesis of nanomaterials include algae, bacteria, fungi and plant extracts. Among known green methods for the production of metal and metal oxide nanomaterials, the use of plant extracts in ultrasonic-assisted green synthesis is a relatively simpler and easier to handle method to produce a better yield of nanomaterials [3]. *Psidium guajava* is a part of the *Myrtaceae* family, which is familiarly known as guava. This small tropical tree has been widely used in medical applications to treat diarrhea, gastrointestinal disorder, cold and swelling [4]. *Psidium guajava* leaf extract possesses metabolic activities, such as antioxidant, antimicrobial, antidiabetic and anticough properties, which are useful in medicine [5,6]. The phytochemicals of guava leaf extract include flavonoid and squalene. The extract of guava leaves contains all such compounds, which are important for the synthesis of nanoparticles [7].

Metallic nanoparticles possess antimicrobial properties for which they are being considered as antibiotics [7]. The green synthesis of TiO_2_, ZnO, MgO, CaO and AgO nanoparticles is gaining importance among the research community [8]. ZnO is a metal oxide n-type semiconductor with a large energy band gap of 3.37 eV. At the nanoscale, ZnO possesses exceptionally beneficial physicochemical properties for highly sophisticated and high-tech applications. The suitable band gap and excitation binding energy are responsible for their excellent properties, such as better photocatalytic activity, UV radiation protection, anti-inflammatory action, fast wound healing and better optical characteristics. The need for robust, highly efficient and durable photocatalysts for the degradation of organic dyes makes ZnO nanoparticles a highly reliable photocatalyst. The UV resistive property of ZnO nanoparticles makes them a very attractive additive for cosmetics and sunscreens. The antimicrobial properties of ZnO nanoparticles are a feasible solution to prevent virulent diseases. Moreover, ZnO nanoparticles also find applications in the biomedical sector [3]. Among various antimicrobial agents, silver and ZnO nanoparticles are said to have the best properties to stop the growth of microorganisms [9,10].

When it comes to the application of nanomaterials, nanotechnology has found its uses in the textile industry due to the growing requirement of durable, antimicrobial and UV protective textiles. Self-cleaning, UV protection, antibacterial activity and mechanical strength can all be achieved by coating nanoparticles on the fabric’s surface [10]. Microorganisms can easily grow on the textile due to natural chemical components present in fiber, which can cause issues, such as stains, bad odor and may affect the strength of the textile. For this reason, textiles with antimicrobial effects are of great concern to keep the human body safe and healthy. Because there are very few functional groups on the surface of the fabric, more functional groups must be created in order to improve nanocoating binding and stability. Nonthermal plasma treatment is one of the chemical and physical approaches that have been developed to boost the binding and adherence of nanoparticles to the fabric. Plasma can improve the surface functionality of textiles, such as wettability, printability, adhesion of coatings, dyeing, desizing and many others without affecting its bulk properties [11,12]. The adhesion of nanoparticles to the fabric surface can be enhanced by imparting polar functional groups through plasma exposure [13]. Noman et al. [12] proposed the sonochemical synthesis of ZnO and their optimization for self-cleaning activities. The maximum color difference (ΔRGB = 99) was obtained for methylene blue. 

Mostly, the wastewater of the textile industries consists of a cationic dye called methylene blue. In developing countries, industrial water is discharged into the open environment without any proper treatment [14]. The dye-containing water not only harms the water bodies and aquatic life but also deteriorates human health. Therefore, it is necessary to find some practically viable methods of degrading the organic dye waste from the textile industry. Therefore, methylene blue is taken as a model target pollutant in this study. Nosocomial infections are illnesses that are acquired during hospitalization or in a hospital setting. The gram-negative Escherichia coli (*E. coli*) is a nosocomial pathogen that spreads infections of the urinary tract and enterocolitis. On the other hand, the gram-positive Staphylococcus aureus (*S. aureus*) causes etiological infection, which is one of the reasons for the significant rate of mortality and morbidity. According to the report of the Broad Institute, 17.3% of clinical infections are caused by *E. coli* and 18.8% are due to *S. aureus* [15]. This study aims to prepare and coat ZnO nanoparticles onto plasma-pretreated cotton fabric through an ultrasonic homogenizer bath in a one-pot sonochemical preparation arrangement. The effect of plasma activation on nanoparticles’ adherence to the fabric surface was examined. The process parameters, such as plasma activation time, amount of ZnCl_2_ and sodium hydroxide were varied to optimize the synthesis conditions for the self-cleaning property of the raw cotton.

## 2. Materials and Methods

### 2.1. Materials

For the experimental work, 100% cellulose cotton was supplied by the National Textile University, Faisalabad. Zinc chloride (ZnCl_2_) and sodium hydroxide (NaOH) of Merck grade and methylene blue were bought at a local science market. Samples of cotton with dimensions 10 × 10 cm^2^ were produced and desized. Prior to DBD plasma activation, the cotton pieces were desized for 1 h in water at 80 °C with a wetting agent (2 g/L), enzyme (3–5 g/L) and sodium chloride (2 g/L). This procedure was carried out to remove the impurities, proteins and stains of grease on the fabric. The desized samples were dried and kept in a moisture-free environment for further experimentation.

### 2.2. Complete Composite Design for Statistical Optimization

There are three kinds of design points in a set of CCD. These points include axial points (±α), center points (0) and factorial points (±1). The value of alpha (α) is taken as 1.68 in the case of the three-input parameter design. The quadratic model is fitted in design to find the maxima and minima of a parameter and the influence of curvature and the response of the surface. The different quantities of input parameters, such as ZnCl_2_, NaOH and plasma exposure time, for CCD-based experiments are illustrated in Table 1. The amount of zinc chloride (1.5–18.5 g) and of NaOH (3.3–11.7 g) was used as an input in CCD in Minitab software. The pattern of quantities of ZnCl_2_ and NaOH was adjusted automatically by the software. The experimental results of color difference (ΔRGB) for self-cleaning of all CCD-based experiments and a control experiment are also illustrated in Table 1. Here “M” represents the sample for the control experiment. Equation (1) was considered to quantify the effect of the input parameters on self-cleaning.

(1) $$Y=b_{0}+\sum^{\;}b_{i}X_{i}+\sum^{\;}b_{i.j}X_{i}X_{j}+\sum^{\;}b_{i.i}X_{i}^{2}\;\;\;\;\;\;\;i\geq j,\;\;i,j=1,2,3$$

In this equation, *b*_0_ is a coefficient of a constant term and *b_i_* is the coefficient of a linear term, while *b_i.j_* and *b_i.i_* express the coefficients of two factors’ interaction and quadratic terms, respectively [13].

### 2.3. Configuration of Nonthermal Plasma

**Figure 1:**
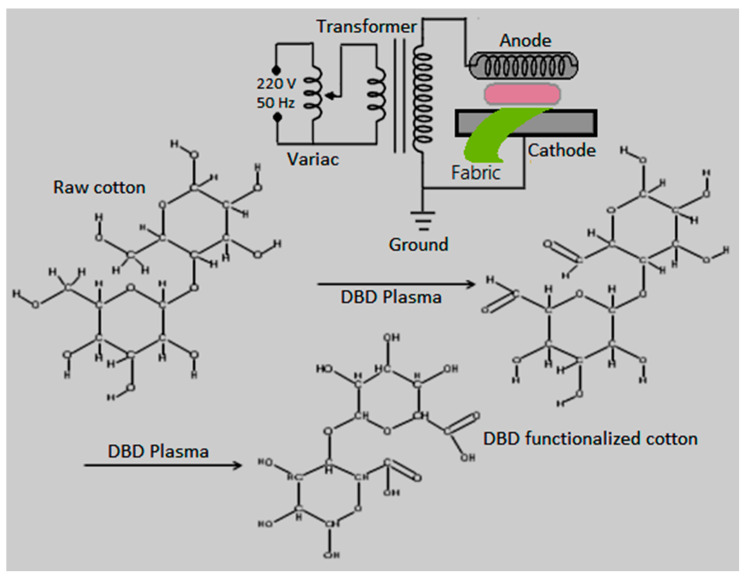
Illustration of the nonthermal DBD system for the activation of cotton and possible chemistry involved in surface functionalization.

The configuration of a nonthermal plasma system (dielectric barrier discharge) involved a grounded movable cylindrical aluminum electrode and a cylindrical anode of copper. As shown in Figure 1, the copper electrode was covered with a 1.5 mm dielectric layer of glass. The gap between electrodes is filled with micro-discharges or filaments when a high voltage or frequency is applied across the electrodes. In this work, a 30 kV transformer was used to produce DBD micro-discharges between electrodes. A dielectric layer between electrodes was used to limit the flow of current across the electrodes and to protect the circuit from breakdown or gas sparking. The dielectric also limits the heating effect due to the formation of a displacement current. The main advantage of DBD is its large electron density, which leads to the uniform surface treatment of textiles [16].

The desized samples were pasted on a moveable cylindrical electrode with paper tape. The movable electrode was rotated at 100 rpm by an electric motor. The DBD system was operated in the open air with an electrode gap of 2 mm at room temperature. At an input voltage of 140 V, the output voltage and current were recorded as 26 kV and 3.80 mA, respectively. The cotton pieces were plasma-activated for different treatment times by following the statistical design in Table 1.

### 2.4. Guava Plant Extract and Synthesis of ZnO Nanoparticles 

Guava is a small tree or shrub of the *Myrtaceae* family. Guava leaves are a popular medicine for ulcers, wound dressing, rheumatic pain and diarrhea [17]. The leaves of guava also have anticancer, antibacterial and anti-inflammatory properties [17]. Guava is chosen in this research work due to the presence of polyphenols (caffeic acid, elligic acid, ferulic acid, gallic acid), flavonoids (kaemp ferol, quercetin), carotenoids (lutein, rubixanthin, phytofluene, lycopene, nechrome) and triterpenes (uvaol, ursolic acid, oleanolic acid) in the crude extract. In the synthesis of ZnO nanoparticles, the functional groups of the aforementioned bioproducts can coordinate Zn (II) and help in the process of stabilization [18]. Nechrome and β-carotene are used in the guava extract work as chain-breaking antioxidants. Therefore, the extract of guava can be used as a biocatalyst/cheating agent for the synthesis of ZnO nanoparticles from a zinc chloride precursor [19].

The guava leaves were thoroughly washed with deionized water multiple times to remove any kind of dust particles, wax and other impurities. The washed leaves were immersed in acetone to remove the green pigments. The leaves were dried in a shady area in a room for 5 to 6 days. The dried leaves were ground in a grinding machine to get a fine powder. Then 30 g of leaf powder was mixed with 300 mL methanol and kept in the dark for 2 days. After that, the solution was placed on a magnetic stirrer at 70 °C for 2 h. The solution was then filtered using Whatman No. 1 filter paper to get leaf extract, which was directly used for the synthesis of ZnONPs.

Different amounts of ZnCl_2_ and NaOH, based on CCD statistical design, were mixed with 100 mL of guava extract to prepare the solutions. To reach equilibrium, each solution was kept in the dark for 12 h. During each experiment, the plasma-activated cotton was dipped completely in the solution in a beaker. The solution and plasma-activated sample together in a beaker were placed in an ultrasonic bath to perform sonication for 75 min at 40 kHz and 80 °C solution temperature [20]. During the sono-synthesis of ZnONPs, the sonic cavitation events create transient high pressure and temperature localized hot zones or bubbles. In the sonolysis of the solution, variations in temperature and pressure of the sono-generated bubbles break the water molecules into $H^{.}$ and ${\mathrm{OH}}^{.}$ radicals. Hydrolysis of precursors and polycondensation of byproducts are made possible by such free radicals, resulting in nanoparticles. These particles bind to the fabric surface through chemical bonding with the functional groups induced on the cotton during plasma processing.

**Figure 2:**
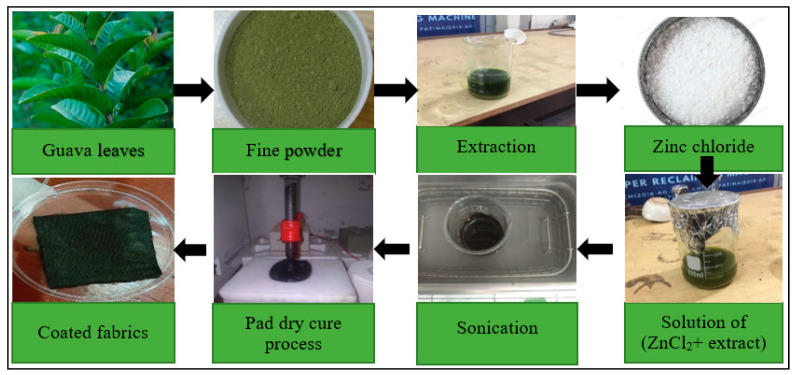
Illustration of steps involved in the one-pot production of ZnO and coating over plasma-activated cotton fabric.

After the sonication, the cotton pieces were taken out and padded in a dry-pad machine to improve the stability on the cotton surface. The whole ZnONPs synthesis and coating procedure is illustrated in Figure 2. The padded sample was washed with distilled water to remove extra particles, which could not make firm contact with the fabric. The fabric pieces were dried to pass them through another cycle. The above-discussed procedure is called one complete coating cycle. Similarly, all the other experiments in Table 1 were conducted by following the CCD design. The optimum and control samples were coated for up to five coating cycles in order to elaborate and compare the self-cleaning, antimicrobial and UV protection traits.

### 2.5. Characterization of Samples

The results concerning the morphology and topography of the control, plasma-treated and optimum samples were observed by scanning electron microscopy (SEM) images. An X-ray diffractometer was used to observe the XRD patterns of the optimum and control samples. An X-ray diffractometer with Cu-Kα radiation of λ = 0.15406 nm was used to collect the XRD patterns and study of the structure of ZnONPs. The obtained results were compared with a standard powder diffraction file (PDF:89-7102). The size of the grains was obtained using Scherrer’s crystallite equation:

(2) $$size\;of\;crystallite\;\left(D\right)=\frac{K\lambda }{\beta cos\theta }\;$$

where *D* shows the size of crystallite, λ is X-ray wavelength, β represents the full width at half maximum and K is the shape constant with a value of 0.89. To validate the formation of ZnO nanoparticles, a UV spectrometer was used to measure the UV-vis spectrum of a solution containing the optimum sample. The functional changes in the plasma-assisted nanocoated optimum sample were studied using Fourier transform infrared spectroscopy. Moreover, the presence of necessary reducing agents in guava extract was confirmed by FTIR analysis. For the study of the adhesion of ZnO nanoparticles towards cotton fabric, the electrostatic surface interaction was examined by the Zeta potential of cotton fabric. Both plasma-activated and blank cotton pieces were analyzed for the zeta potential measurements [21].

### 2.6. Photocatalytic Activity of Nanocoated Samples

UV light irradiations were irradiated on ZnO-coated cotton samples to investigate the photodegradation of methylene blue dye from the samples. For this purpose, all ZnO-coated samples were immersed separately in 0.01% (*w/v*) solutions of MB dye. The developed sample in MB solution was kept in the room for 6 h to reach equilibrium. Then the developed sample was drawn out from the MB solution and allowed to dry at ambient temperature. The dried sample was exposed to UV 500 W xenon lamp for 6 h. A distance of 30 cm was kept between the sample and the UV lamp. The methylene blue-stained samples were placed under a UV lamp because the energy of the emitted UV-A light (λ=320–400 nm) is comparable to the band gap (3.37 eV) of ZnO NPs coated on cotton. The color differences (ΔRGB) of all developed samples were observed by image processing in MATLAB software. Equation (3) was used to determine the color differences of UV irradiated samples and without UV irradiated samples:

(3) $$∆\mathrm{RGB}=({\left(R_{2}-R_{1}\right)}^{2}+{\left(G_{2}-G_{1}\right)}^{2}+{\left(B_{2}-B_{1}\right)}^{2}$$

where $R_{1}G_{1}B_{1}$ are the color coordinates without UV irradiation of the sample and $R_{2}G_{2}B_{2}$ are the color coordinates of the UV irradiated sample.

### 2.7. Radical Scavenger Analysis 

The photocatalytic mechanism was further studied by detecting the reactive species, such as holes and radicals, in radical scavenging experiments. The superoxide radical ($O_{2}^{-}$), holes (h^+^) and hydroxyl radical (OH) are trapped by mixing tertbutanol (t-BuOH) (OH scavenger), ammonium oxalate (AO) (h^+^ scavenger) and p-benzoquinone (P-BQ) ($O_{2}^{-}$) scavenger into the solution of reaction, respectively. Typically, 10 mM of radical scavenger and 10 mg ZnO were put in a 50 mL dye solution of (30 mg/L) concentration. Then the suspension was placed under the UV lamp for an equal time. The role of active radical species was determined by calculating the removal rate of the dye.

### 2.8. Antimicrobial and UV Protection Activity

For the antibacterial test, the agar disc diffusion method was used. The bacteria culture weight for nutrient agar of 28 g was prepared and dissolved in 1000 mL of distilled water in Petri plates. The borer was autoclaved for 15 min, and then the media was cooled. The E. Coli bacteria and S. Aureus were then added to the plates and incubated for 24 h at 37 °C to check the inhabitation zones of optimal and control samples [22]. 

UV protection characteristics of optimized and control samples were investigated further. The AATCC TM 183 standard was used to conduct the transmittance tests and to calculate the UV protection factor (*UPF*). The following equation was used to compute *UPF*:

(4) $$UPF=\frac{\sum_{\lambda =280}^{400}E_{\lambda }.S_{\lambda }.∆\lambda }{\sum_{\lambda =280}^{400}E_{\lambda }.S_{\lambda }.T_{\lambda }.∆\lambda }\;\;$$

where $S_{\lambda }$ is the solar spectral irradiance, $E_{\lambda }$ is the relative erythemal spectral effectiveness, Δλ is the measured wavelength interval and $T_{\lambda }$ is the spectral transmittance of the specimen [23].

## 3. Results and Discussion

### 3.1. Mechanism of ZnO Coating of Cotton

**Figure 3:**
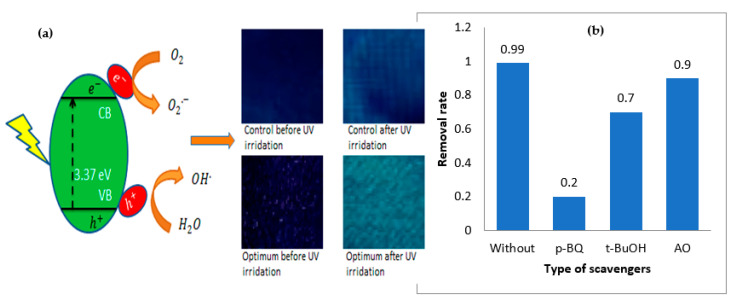
(**a**) Mechanism of photodegradation of MB on ZnO-coated fabric with a color difference of optimum and control samples before and after 6 h of UV irradiation and (**b**) removal rate of methylene blue in the presence of different scavengers.

The photocatalytic reaction usually consists of surface oxidation, photoexcitation, charge migration and separation. It is critical to determine which reactive species are most important in dye degradation. Figure 3b shows the rate of degradation both in the absence and presence of the scavengers. In photocatalytic degradation of methylene blue, there was a slight change in t-BuOH and AO. There was a considerable reduction (18.67) in the removal rate of methylene blue in the presence of p-BQ scavenger. The addition of benzoquinone showed the highest reduction in the removal rate, followed by tert-butanol and ammonium oxalate. These findings agree well with the study of Huang et al. [24]. They used similar scavengers and conditions to study their effect on the dye removal rate. The superoxide radical was discovered to be the most important reactive species during the photodegradation of methylene blue. 

The alkaline environment plays a central role in the formation and coating of ZnONPs on the cotton samples. The cotton cellulose was modified to cellulosate anion in an alkali medium, which creates extra sites for the adsorption of zinc ions, as illustrated in the reaction in Equation (5). Positively charged zinc ions adsorb onto the surface of fibers of cellulose due to electrostatic attraction, which further promotes the synthesis of ZnONPs [23]. The cellulosic fabric surface provided space for the nucleation and growth of ZnO during sonication at 80 °C. The acoustic cavitation phenomenon during sonication causes the growth of shortly lived high pressure and temperature zones or bubbles. These bubbles burst with a rise in pressure and temperature and transfer their energy to the medium, where they produce $H^{.}$ and ${\mathrm{OH}}^{.}$ radicals by breaking water molecules. The free radicals initiate the precursor hydrolysis and poly-condensation of the products into nanoclusters, as produced nanoparticles bind to the fabric surface through chemical bonding with the functional groups induced on the fabric surface during plasma treatment. In this synthesis process, guava extract works as a reducing and stabilizing agent for ZnO nanoparticles [25]. In an alkaline medium, zinc hydroxide reduces to Zn^2+^ species in the hydroxylation process, which further reduces to ZnO nanoparticles during the heating of fabrics at 80 °C through the following reactions:

(5) $$\mathrm{Cell}-\mathrm{OH}\overset{\mathrm{Alakli}\;\mathrm{medium}}{\to }\;\mathrm{Cell}-O^{-}$$

(6) $$\mathrm{NaOH}\to {\mathrm{Na}}^{+}+{\mathrm{OH}}^{-}$$

(7) $${\mathrm{ZnCl}}_{2}\to {\mathrm{Zn}}^{2+}+2{\mathrm{Cl}}^{-}$$

(8) $${\mathrm{Zn}}^{2+}+{\mathrm{OH}}^{-}\to \mathrm{Zn}{\left(\mathrm{OH}\right)}_{2}$$

(9) $$\mathrm{Zn}{\left(\mathrm{OH}\right)}_{2}\;\to \mathrm{ZnO}$$

### 3.2. Photocatalytic Activity of ZnO-Coated Cotton 

The photocatalytic activity of ZnO coated cotton was checked by evaluating the degradation of MB on its surface under the irradiation of ultraviolet light. The results of self-cleaning (ΔRGB) for plasma-assisted nanocoated and control samples are shown in Table 1. The self-cleaning efficiency of sample 5 was found to have a maximum of (ΔRGB = 100.4) among the other plasma-assisted coated samples and control samples (ΔRGB = 94.2). This sample was taken as the optimum one for further experiments. The optimum conditions of input parameters in this research were the amount of ZnCl_2_ (17.5 g), NaOH (11.7 g) and plasma exposure time (70.2 s). The high self-cleaning value of the control sample ‘M’ is due to the fact that it was subjected to the same experimental conditions as the optimized sample, with the exception of plasma treatment. Moreover, the acoustic cavitation phenomenon can produce a temporary high pressure and temperature localized hot zones during the sono-synthesis of nanoparticles. A change in pressure and temperature can generate $H^{.}$ and ${\mathrm{OH}}^{.}$ radicals in the sonolysis of $H_{2}O.$ Such free radicals enable the hydrolysis of precursors and poly-condensation of byproducts to nanoparticles. The trapped air between the inter and intra-yarns serves as a nucleus for the formation of bubbles in the fabric. Therefore, powerful convection produced by the motion of the transient bubbles in close proximity to the fabric intensifies the fabric’s mass transfer. As a result, the flow of reactional fluid can be accelerated through the fabric, resulting in stronger adsorption of nanoparticles. Moreover, nanoparticles have a large affinity for hydroxyl groups, and the dehydration reaction between the hydroxyl groups of both nanoparticles and fabric cause the formation of interfacial bonding that result in better adhesion of nanoparticles on the surface of fibers [26]. The difference in colors of optimum and control samples is shown in Figure 3b. The results revealed that DBD plasma treatment increases the self-cleaning efficiency of ZnONPs. In photocatalytic degradation mechanism, the photocatalytic reaction consists of photoexcitation, separation of charges and their migration, and then oxidation and reduction reactions at the surface [27]. The irradiation of UV generates reactive species $h^{+},{\;\mathrm{OH}}^{-}{\;\mathrm{and}\;O}_{2}^{.-}.$ The mechanism of photodegradation of MB is shown in Figure 3a. The corresponding UV energy is larger than the band gap of ZnO (3.37 eV), so it can promote the generation of electrons and holes. The energy of UV irradiations transfers the valence electrons to the conduction band. The UV-generated holes ($h^{+}$) directly react with H_2_O or hydroxyl (${\mathrm{OH}}^{-}$) groups to create hydroxyl radicals (${\mathrm{OH}}^{.-}$). The surface oxygen reduces to superoxide radical ($O_{2}^{.-}$) by photoelectrons. Finally, MB decomposes by ${\mathrm{OH}}^{.-}$ and $O_{2}^{.-}$ radicals by following the reactions given below:

(10) $$\mathrm{ZnO}+hv\to h^{+}+e^{-}$$

(11) $$h^{+}+{\mathrm{OH}}^{-}{\;\mathrm{or}\;H}_{2}O\to {\mathrm{OH}}^{.}$$

(12) $$e^{-}+O_{2}\to O_{2}^{-}$$

(13) $${\mathrm{OH}}^{-}{\mathrm{and}\;O}_{2}^{.-}+\;\mathrm{MB}\to {\mathrm{CO}}_{2}+H_{2}O$$

Noman et al. [12] sonochemically synthesized ZnO nanoparticles for self-cleaning application. ZnCl_2_ and NaOH were used as precursor and reaction accelerators in the synthesis of nanoparticles. The maximum self-cleaning (ΔRGB = 99) value was obtained for the degradation of the methylene blue solution. In this research, the maximum self-cleaning of ΔRGB = 100.4 was possible, which is higher than the value reported by Noman and co-workers.

### 3.3. Statistical Optimization and Analysis of Variance

The influence of input parameters on response parameters (self-cleaning) was evaluated by studying the experimental CCD design, response surface plots and contour plots. The relationship between the input parameters and output response was studied by mathematical modeling Equation (14). This equation can be used to find the percentage degradation of MB with coded variables as: A = amount of ZnCl_2_, B = amount of NaOH, C = DBD plasma exposure time and Y = self-cleaning (ΔRGB).

Y = 37.7 + 3.054 (A) − 0.11 (B) + 0.586 (C) − 0.0313 (A × A) − 0.0360 (B × B) − 0.00619 (C × C) − 0.0913 (A × B) − 0.00230 (A × C) + 0.0434 (B × C)(14)

Table 2 shows the derived ANOVA findings for the quadratic model. The linear and interaction factors (*p* < 0.05) significantly influence the degradation of MB. In other words, the model shows the effect of input parameters on the response. The large F-value indicates that the model variance is greater than the random error. The prediction of the model for experimental value is very good, as the F-value of this model is 26.72. This result proves that the prediction strength fitted well with the model. The R-Squared coefficients can be used to describe the model’s fit as well. The high values of R-Square and R-Square (adj) revealed that MB deterioration is significant when the solution is exposed to UV radiation. The values of input parameters, which influenced the dye removal most, are 96% ZnCl_2_ and 0.125% NaOH. The Pareto analysis revealed that plasma treatment time is also an effective parameter in this study [25].

**Figure 4:**
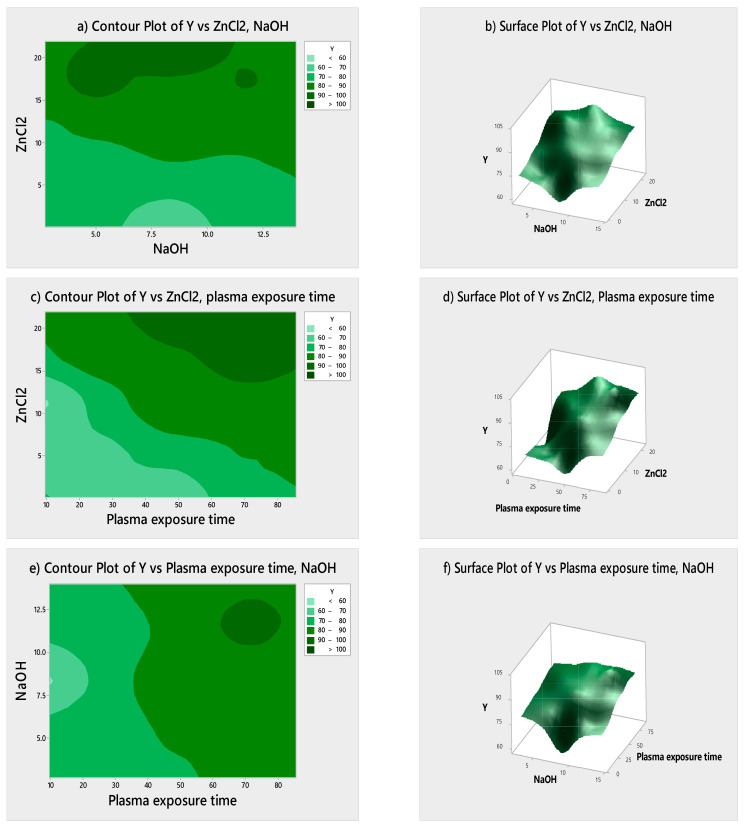
The surface response and contour plots of MB degradation for different experimental input parameters: (**a**,**b**) ZnCl_2_ vs. NaOH, (**c**,**d**) ZnCl_2_ vs. plasma exposure time and (**e**,**f**) NaOH vs. plasma exposure time.

The surface contour plots were produced to analyze the influence of input parameters on the output parameter for optimization of the coating process. Figure 4 shows the contour and response surface graphs. One parameter was kept constant in contour plots while the other two parameters were varied. Figure 4a,b shows that an increase in the concentration of ZnCl_2_ had a different influence on MB degradation, which depends on the concentration of NaOH. For a large concentration of ZnCl_2_, MB degradation was low and vice versa. The MB degradation increased with an increase in NaOH and was found to be the maximum when NaOH was in the range of 3–11 g. From Figure 4c,d, MB degradation increased with an increase in the concentration of both ZnCl_2_ and plasma exposure time, but it was at its maximum for higher values of both input parameters. Similarly, Figure 4e,f shows the effect of NaOH and plasma activation time on MB degradation. Dye degradation increased as both input parameters were increased [16].

**Figure 5:**
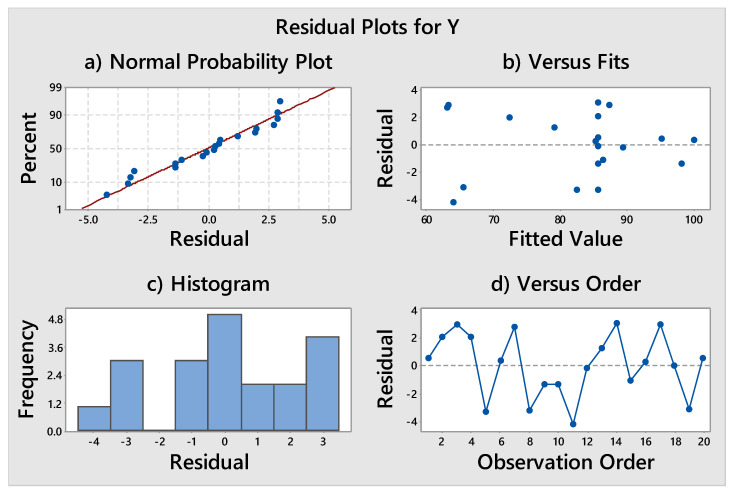
Graphs of normal residuals, (**a**) percent vs. residual, (**b**) fitted value vs. residual, (**c**) frequency vs. residual and (**d**) residual vs. observation order.

The model’s suitability was determined by plotting residual graphs, as shown in Figure 5. The response for MB degradation percentage followed the normal distribution in Figure 5a. It indicates that there is no obvious problem with normalcy and that no response transformation is required. The non-systematic behavior of the plot in Figure 5b implies that the variance of the original data remains constant for each value of the response. Similarly, the histogram of all observations in Figure 5c shows that the residuals are regularly distributed. In conclusion, all plots in Figure 5 show that the model is adequate for the photocatalytic removal of MB from the fabric surface.

### 3.4. XRD Analysis

**Figure 6:**
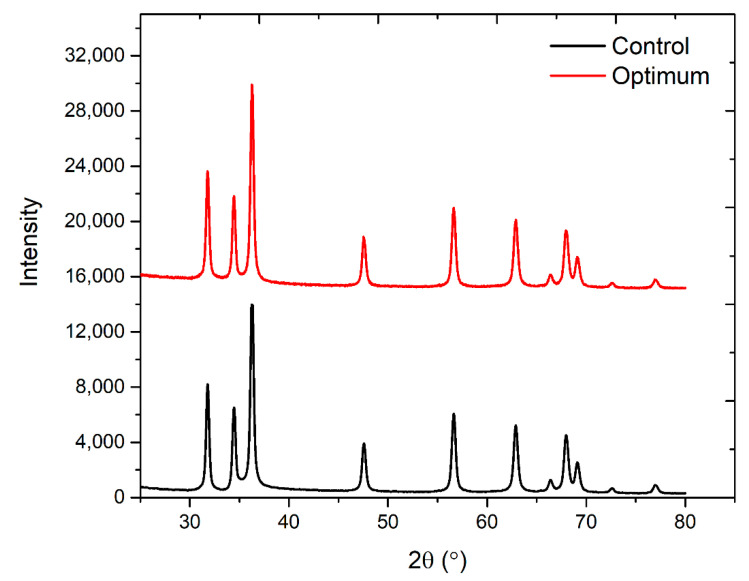
XRD patterns of control and optimum cotton samples after five coating cycles.

The XRD patterns of optimum and control ZnO-coated cotton are presented in Figure 6. The structural properties of the developed sample were examined through XRD analysis. From the XRD profile, the diffraction peaks showed the formation of (100), (002), (101), (102), (110), (103), (112), (201) and (004) planes at 2θ of 31.54°, 34.40°, 36.71°, 47.45°, 56.36°, 62.82°, 67.67°, 70.13° and 71.3°, respectively. This analysis confirmed the polycrystalline nature of the coated ZnO nanoparticles. Table 3 presents the positions (2θ) along with other XRD parameters. A distance of 2.477 Å was found between the planes of the lattice. The relative intensity of peak (2θ=34.40°) was sharp and had a higher intensity for the optimum sample, which indicates a higher quantity of ZnO nanoparticles compared to the control sample (Figure 6). The average size measured from the Scherrer equation was found to be 41.34 nm. The standard XRD characteristic peaks revealed the hexagonal wurtzite structure of ZnO. The other peaks at 2θ=15−25° showed the crystalline nature of cellulose in cotton fabric. The XRD characteristic peaks matched well with JCPD file card No. 36-1451. The ZnO coating on the optimum sample was found denser since the intensity of peak (002) is larger compared to the control sample. 

### 3.5. SEM Analysis

**Figure 7:**
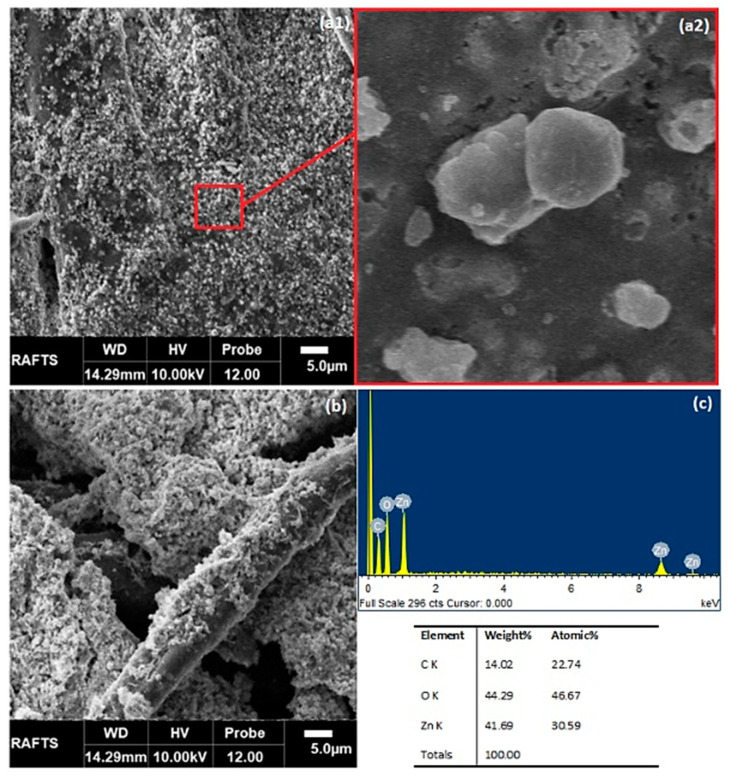
SEM images of (**a1**) optimum sample after five coating cycles, (**a2**) highly magnified image of the selected zone, (**b**) control cotton sample after five coating cycles and (**c**) EDX of ZnONPs.

SEM analysis was used to observe the effect of plasma treatment on ZnO coating. The surface of the untreated DBD plasma cotton sample was smooth, and there were no pits and cracks on the surface (Figure 7a). The surface of the DBD plasma-treated sample became rough because plasma treatment induced pits and wades due to the plasma etching effect (Figure 7b). The ZnO coating on the optimum sample was homogeneous and dense. The coating became more and more homogeneous and dense with an increase in the number of coating cycles. The five-times coated optimum sample showed a higher quantity of ZnO on its surface. The ZnO coatings on the control sample remained partially homogeneous and less dense compared to the optimum sample. The plasma-induced functional groups (COOH, OH) had a strong interaction with ZnO nanoparticles, so the nanocoating became denser and more homogeneous. The highly magnified SEM image of ZnONPs on the plasma functionalized surface revealed that the nanoparticles have a well-defined shape. Most of the nanoparticles on the plasma functionalized fabric were hexagonal in shape. It was difficult to define the shape of the nanoparticles on the control sample. The nanoparticles were agglomerated into larger clusters of varying morphologies. The size of the nanoparticles on the plasma-functionalized fabric was measured by using SEM images in ImageJ software. The average particle size was measured to be about 50 nm.

**Figure 8:**
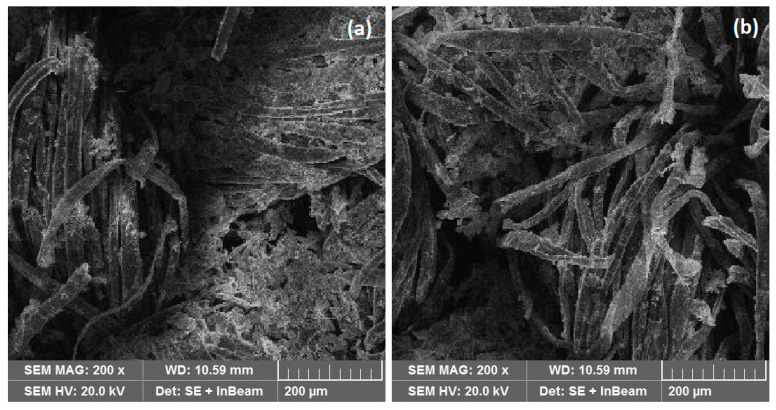
SEM images of the (**a**) optimum sample after five washing cycles and (**b**) control sample after five washing cycles.

The control and optimum samples were subjected to five washing cycles in distilled water to check the stability of the nanoparticles on the fabric. SEM images of both samples were generated to observe the removal of nanoparticles on washing, as shown in Figure 8. The nanoparticles showed good stability on the optimum sample compared to the control. The plasma treatment introduced functional groups for high adhesion of nanoparticles on the control.

### 3.6. UV-Vis Analysis

**Figure 9:**
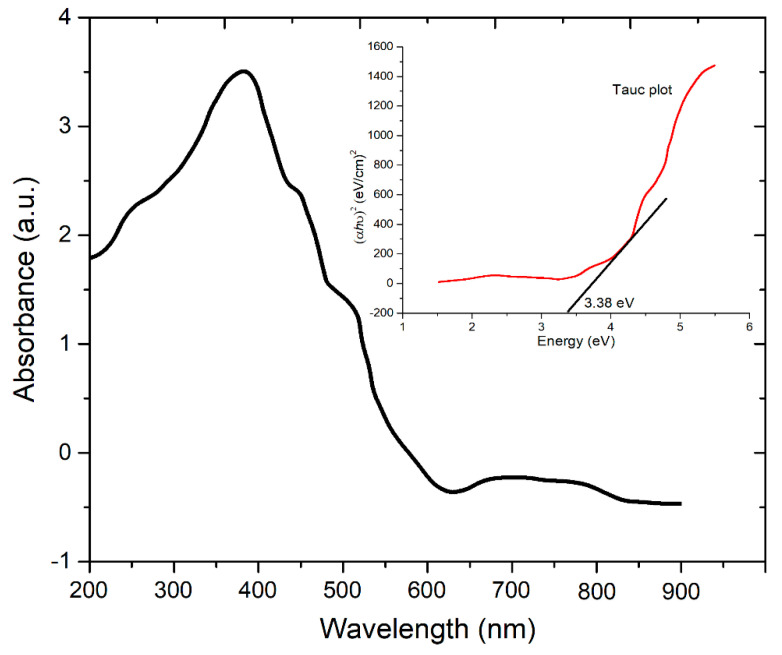
UV-vis spectrum of ZnO suspension of the optimum sample, the inset shows the Tauc plot.

The UV-vis absorption spectrum of ZnO suspension for the optimum sample is shown in Figure 9. The absorption peak around 360 nm revealed that the biosynthesized ZnO nanoparticles had a nano-dimension. The absorption peak confirms the reduction of zinc chloride into ZnO nanoparticles due to the presence of guava extract and an alkali medium. The direct band gap was calculated through the measurement of the slope of the Tauc plot, as shown in the inset. The measured value of the direct band gap was 3.38 eV.

### 3.7. FTIR Analysis

**Figure 10:**
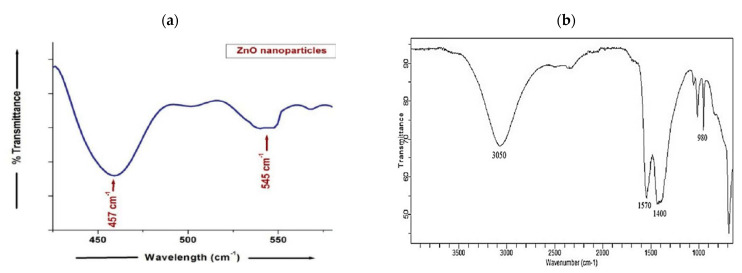
FTIR spectra of the (**a**) optimum sample and (**b**) guava leave extract.

The FTIR spectrum of the ZnO-coated optimum sample is shown in Figure 10. The formation of ZnO nanoparticles was confirmed by the presence of a peak at 545 cm^−1^. The other observed peak at 457 cm^−1^ was due to some impurities of the extract (Figure 10a). The FTIR spectrum of the guava extract identified the functional groups and biomolecules, which were responsible for the efficient synthesis, stabilization and capping of the ZnO nanomaterial. The peaks at 980, 1030, 1400, 1570 and 3050 cm^−1^ were observed, as shown in Figure 10b. The observed peaks were the characteristic of terpenoids and flavanones that were abundant in the guava leaf extract. The peaks at 980 and 1030 (polysaccharide groups), 1400 (nitrosamine), 1570 (diketones) and 3050 cm^−1^ (C–H and carboxyl acid) suggested the presence of terpenoids and flavanones in the extract, which are essential for the formation of nanoparticles.

### 3.8. Antimicrobial Activity of ZnO-Coated Cotton

The antimicrobial activity of nanoparticles is related to their photocatalytic efficiency to produce reaction oxygen species (ROS), such as $O_{2}^{*-}$, H_2_O_2_, and *OH, through oxidation and reduction reactions on the surface of crystals under the irradiation of light. Although the exact mechanism of antimicrobial activity is not entirely understood, it is proposed that when microorganisms come into contact with nanoparticles, the ROS species can penetrate into the cell membrane by diffusion processes and damage the cellular molecules, including lipids, carbohydrates and nucleic acids, leading to the death of the cell. Further, the cell wall can be disrupted by ROS through the process of lipid peroxidation. Moreover, ROS can disrupt the electron transport chain in transmembrane. The small size nanoparticles can penetrate into the cell and cause the generation of photo ROS inside the cell. ROS killing effect has been observed in many types of cells, such as yeasts, viruses, and fungi [28]. The antimicrobial activity of the optimum and control samples was tested against gram-positive and gram-negative bacteria. E. Coli and S. Aureus were used as gram-negative and gram-positive strains, respectively. Table 4 shows the zone of inhibition against both strains for the optimum and control samples, which shows that with an increase in the number of coatings, the zone of inhibition increased. In a comparative study between the optimum and control samples, the results revealed that the zone of inhibition for the optimum sample was larger compared to the control sample. The reason was that the plasma treatment of the optimum sample imparted the important functional groups on the surface, which results in an increased coating quantity of ZnO nanoparticles during the sonication process [22].

Kalpana et al. [28] studied the antibacterial activity of biosynthesized ZnO nanoparticles. The particles were stabilized on cotton fabric by using acrylic binder. ZnO-coated cotton showed the zone of inhibition of about 12 ± 0.23 mm against *S. aurues* and 10 ± 0.78 mm against *E. coli* strains. The results of the presented work are comparable to the aforementioned results. These findings imply that ZnONPs have the potential to produce sterile fabrics for medical treatments and hospital environments where infection is a concern.

### 3.9. Ultraviolet Protection and Zeta Potential

The results of the UV protection factor are presented in Table 5. The protection factor increased with coating cycles of the optimum sample. The transmittance of UVA and UVB rays decreased as the number of cycles were increased from 1 to 5. On plasma treatment, the ZnO nanoparticles were uniformly distributed onto the cotton fabric. When the number of cycles was increased, the quantity of ZnONPs increased, which resulted in a higher protection factor of up to 50 and less transmittance of UVA and UVB rays. It was concluded that ZnO nanoparticles possess strong UV protective properties and can be used as a coating for the production of UV protective cellulose fabrics.

The zeta potential for the optimum and control samples was also measured and compared. The Zeta potential of the control sample was measured to be about 0.97 mV, which decreased to −0.5 mV for the optimum sample. A decrease in zeta potential after plasma treatment showed that the high functional group density exists at the surface of the fabric [19]. Due to plasma-generated functional groups, the zeta potential decreased, which in turn increased the adhesive property of ZnONPs towards cotton fabrics. The adhesion strength of ZnO nanoparticles on the surface of cotton was also studied through SEM images. Both the optimum and control samples were washed up to five times by distilled water. After five washing cycles, SEM images were taken, as shown in Figure 8. SEM analysis revealed that ZnO nanoparticles remained stuck even after five washing cycles. Further, the quantity of ZnONPs on the optimum sample was found to be more compared to the control sample. 

## 4. Conclusions

The self-cleaning property, along with antimicrobial and UV protective properties, of open-air plasma-assisted ZnO-coated cotton fabrics was studied. The extract of guava leaves was used for the synthesis of hexagonal wurtzite ZnO nanoparticles with an average grain size of 41.34 nm because it contains important reducing and capping agents, as confirmed by FTIR analysis. The degradation of methylene blue dye was optimized by RSM-based CCD. The relationship between the removal of MB by ZnONPs and input parameters was developed by using a quadratic polynomial equation with a large determination coefficient (96.01). The optimum degradation (Δ = 100.4) of MB was obtained for optimum conditions of ZnCl_2_ (17.5 g), NaOH (11.7 g) and plasma exposure time (70.2 s). The percentage influence of the plasma treatment was 3.54 for self-cleaning activity. The plasma pre-treatment also enhanced the antimicrobial and UV protective properties of the ZnO-coated sample. The plasma treatment increased the UPF up to 50+ compared to the control sample. Therefore, plasma treatment was considered as an important approach to enhance the adsorption of nanoparticles on textiles for their commercial applications in removing organic dyes and killing bacteria.

## Figures and Tables

**Table 1:** Actual values of input parameters along with the experimental results of all CCD-based experiments and a control experiment.

Sr. No	ZnCl_2_ (g)	NaOH (g)	DBD Plasma Time (s)	Self-Cleaning Value (ΔRGB)
1	17.5	5.0	70.2	95.6
2	11.0	8.4	47.6	87.6
3	17.5	5.0	25.0	90.2
4	4.5	5.0	70.2	74.4
5	17.5	11.7	70.2	100.4
6	11.0	2.7	47.6	79.1
7	4.5	11.7	25.0	65.7
8	11.0	8.4	47.6	82.3
9	21.9	8.4	47.6	96.8
10	11.0	8.4	47.6	84.2
11	11.0	8.4	9.6	59.7
12	11.0	8.4	85.6	89.1
13	17.5	11.7	25.0	80.3
14	11.0	8.4	47.6	88.6
15	11.0	13.9	47.6	85.3
16	4.5	11.7	70.2	85.6
17	4.5	5.0	25.0	66.1
18	11.0	8.4	47.6	85.5
19	0.1	8.4	47.6	62.4
20	11.0	8.4	47.6	86.1
M	17.5	11.7	0.0	94.2

**Table 2:** Summary of ANOVA analysis used to study self-cleaning of the coated cotton.

Source	DF	Adj. SS	Adj. MS	F-Value	*p*-Value	Significance
Model	9	2362.48	262.50	26.72	0.001	Significant
Linear	3	2084.62	694.87	70.72	0.000	Significant
ZnCl_2_	1	1286.57	1286.57	130.94	0.000	Significant
NaOH	1	19.04	19.04	1.94	0.194	Not Significant
Plasma exposure time	1	779.01	779.01	79.29	0.000	Significant
Square	3	158.88	52.96	5.39	0.018	Significant
(ZnCl_2_) ^2^	1	25.23	25.23	2.57	0.040	Significant
(NaOH) ^2^	1	2.35	2.35	0.24	0.635	Not Significant
(Plasma exposure time)	1	144.05	144.05	14.66	0.003	Significant
2-Way Interaction	3	118.97	39.66	4.04	0.040	Significant
ZnCl_2_ × NaOH	1	31.60	31.60	3.22	0.103	Not Significant
ZnCl_2_ × Plasma time	1	0.91	0.91	0.9	0.767	Not Significant
NaOH × Plasma time	1	86.46	86.46	8.80	0.014	Significant
Error	10	98.25	9.83			
Lack of Fit	5	72.23	14.45	2.77	0.144	
Pure Error	5	26.03	5.21			
Model Summary	R-sq = 96.01%, R-sq (adj) = 92.41%					

**Table 3:** XRD parameters of ZnO after five coating cycles.

2*θ*	h k l	d-Spacing (Å)	FWHM
31.54°	(100)	2.814	0.39
34.40°	(002)	2.601	0.17
36.71°	(101)	2.471	0.46
47.45°	(102)	1.911	0.40
56.36°	(110)	1.624	0.60
62.82°	(103)	1.477	0.36
67.67°	(112)	1.377	0.50
70.13°	(201)	1.342	0.30
71.3°	(004)	1.312	0.29

**Table 4:** Zone of inhibition of control and optimum cotton samples against gram-positive and gram-negative bacteria.

Zone of Inhabitation against Gram-Positive Bacteria			Zone of Inhabitation against Gram-Negative Bacteria	
Control Sample		Optimum Sample	Control Sample	Optimum Sample
Coating Cycles	Inhabitation Zone	Inhabitation Zone	Inhabitation Zone	Inhabitation Zone
1	1.90 mm	3 mm	1.95 mm	3.1 mm
3	2 mm	6 mm	2.1 mm	6.3 mm
5	3 mm	10 mm	2.98 mm	11 mm

**Table 5:** Ultraviolet protection factor (UPF), transmission in UVA and UVB region and the UPF rating of the cotton samples.

Samples	UPF	UV Transmittance (%)		UPF Rating
		UV-A (315–400 nm)	UV-B (280–315 nm)	
Five time coated (control)	5.7	29.27	26.84	Unrelatable
One time coated (optimum)	33.56	7.43	2.36	30
Three time coated (optimum)	58.22	6.71	1.93	50+
Five time coated (optimum)	69.87	6.41	1.76	50+
